# Wheat-Maize Intercropping With Reduced Tillage and Straw Retention: A Step Towards Enhancing Economic and Environmental Benefits in Arid Areas

**DOI:** 10.3389/fpls.2018.01328

**Published:** 2018-10-12

**Authors:** Wen Yin, Yao Guo, Falong Hu, Zhilong Fan, Fuxue Feng, Cai Zhao, Aizhong Yu, Qiang Chai

**Affiliations:** ^1^Gansu Provincial Key Laboratory of Aridland Crop Science, Lanzhou, China; ^2^College of Agronomy, Gansu Agricultural University, Lanzhou, China; ^3^College of Water Conservancy and Hydropower Engineering, Gansu Agricultural University, Lanzhou, China

**Keywords:** carbon emission, crop productivity, economic benefits, strip intercropping, reduced tillage, straw retention

## Abstract

Intercropping is considered a promising system for boosting crop productivity. However, intercropping usually requires higher inputs of resources that emit more CO_2_. It is unclear whether an improved agricultural pattern could relieve this issue and enhance agricultural sustainability in an arid irrigation area. A field experiment using a well-designed agricultural practice was carried out in northwest China; reduced tillage, coupled with wheat straw residue retention measures, was integrated with a strip intercropping pattern. We determined the crop productivity, water use, economic benefits, and carbon emissions (CEs). The wheat-maize intercropping coupled with straw covering (i.e., NTSI treatment), boosted grain yield by 27–38% and 153–160% more than the conventional monoculture of maize and wheat, respectively, and it also increased by 9.9–11.9% over the conventional intercropping treatment. Similarly, this pattern also improved the water use efficiency by 15.4–22.4% in comparison with the conventional monoculture of maize by 45.7–48.3% in comparison with the conventional monoculture of wheat and by 14.7–15.9% in comparison with the conventional intercropping treatment. Meanwhile, NTSI treatment caused 7.4–13.7% and 37.0–47.7% greater solar energy use efficiency than the conventional monoculture of maize and wheat, respectively. Furthermore, the NTSI treatment had a higher net return (NR) by 54–71% and 281–338% and a higher benefit per cubic meter of water (BPW) by 35–51% and 119–147% more than the conventional monoculture of maize and wheat, respectively. Similarly, it increased the NR and BPW by 8–14% and 14–16% in comparison with the conventional intercropping treatment, respectively. An additional feature of the NTSI treatment is that it reduced CEs by 13.4–23.8% and 7.3–17.5% while improving CE efficiency by 62.6–66.9% and 23.2–33.2% more than the conventional monoculture maize and intercropping treatments, respectively. We can draw a conclusion that intercropping maize and wheat, with a straw covering soil surface, can be used to enhance crop production and NRs while effectively lowering CO_2_ emissions in arid oasis irrigation region.

## Introduction

The gap between food demand and grain production has become larger in recent years because of the continuously increasing demand from a growing population (Godfray et al., 2010). Arable lands are limited on the planet, and these areas have been declining year by year because of urbanization, especially in northwestern China. Therefore, the need for the identification of methods to produce more grain using the limited arable land is a very urgent issue. The attainment of high crop yields on the existing lands in developing countries is of great importance to meet this goal with minimal environmental impacts (Tilman et al., 2011). Crop cultivation in many such areas, however, typically makes use of the conventional agricultural practices that cause serious soil degradation, water erosion (Chen et al., 2010), and emission of greenhouse gasses into the atmosphere (West et al., 2010). Water shortages threaten agricultural sustainability in the arid and semiarid regions. For example, in the Hexi arid oasis region of northwestern China, the main grain producing area, the annual precipitation during the crop growing season is between 50 and 150 mm, while the annual potential soil water evaporation is typically greater than 2400 mm, causing the agricultural production to rely largely on underground water for irrigation (Yin et al., 2015). However, the overexploitation of groundwater has caused some aquifers to shrink. The amount of available freshwater for agriculture has decreased, and crop production has been seriously threatened (Brown and Halwell, 1998). Furthermore, high-input farming systems of densely populated regions have been confirmed to increase the cost of production (Garnett et al., 2013). The greater agricultural production inputs (such as inorganic fertilizers, plastic film, pesticides, and water) are causing the carbon emissions (CEs) from farmlands to have a negative impact on the environment (Challinor et al., 2014), thus, producing more greenhouse gasses (Burney et al., 2010). Therefore, efficient tillage and mulching practices are urgently required to satisfy the goals of enhancing crop productivity and improving water utilization while reducing emissions of CO_2_ from farmland ecosystems. A key question is whether we can design an improved farming system that effectively addresses these above issues and, thus, achieve sustainable agriculture in highly populated, natural resource-limited areas.

Intercropping is a systematic approach that makes full use of nutrient and water resources, achieves agricultural biodiversity, and increases yield significantly in comparison with crop monocultures (Qin A. et al., 2013). Intercropping is found to play a crucial role in securing the grain supply and raising the income of farmers in developing countries, thereby, balancing higher food demands and lower water utilization (Chen et al., 2015; Yin et al., 2017). Wheat-maize strip intercropping, an intensive cropping system with high inputs and outputs, was introduced to northwestern China a long time ago. Thus, this system is proven to be productive in oasis regions where climatic conditions allow for only annual, one-season cropping patterns (Qin A. et al., 2013). Previous studies have shown that wheat-maize intercropping can be an effective pattern in balancing grain demand and supply (Qin A. et al., 2013; Gou et al., 2016), reducing global warming potential (Gan et al., 2011a), and improving carbon sequestration (Fang et al., 2010). However, the development of this system has been seriously restricted because of the high consumption of water. Enhancing the productivity and benefits of this system requires more effective options in improving its water use efficiency (WUE) and water productivity (WP). No-tillage and reduced tillage, combined with straw residue, serve as viable options. In brief, reduced tillage and no-tillage approaches are characterized by improvements to soil quality, conservation of soil moisture (Shaver et al., 2002), and increases in soil carbon sequestration (Chatskikh et al., 2008). Crop residue when returned to the soil can help to maintain soil moisture (Yin et al., 2015), sequester carbon (Ghimire et al., 2012) and reduce CEs (Hu et al., 2015). Meanwhile, reduced tillage or no-tillage approaches, combined with straw residue, can suppress weeds, decrease the input of herbicides (Bernstein et al., 2014), reduce the influence of machinery, and decrease the cost of crop production (Garnett et al., 2013). However, although there have been many studies on the sustainability of environmental benefits for natural resources, no academic information is available to determine whether integrating no-tillage and straw retention simultaneously into the same system can enhance the sustainability of the environmental and economic benefits of different crops in intercropping patterns of artificial farmland systems. Comprehensive analysis of the economic and environmental benefits of wheat-maize intercropping is rarely undertaken for reduced tillage coupled with wheat straw residue.

With these issues in mind, we established an innovative farming system, with two key agronomic measures: (i) crop intensification via strip intercropping and (ii) crop residue management approaches combined with reduced tillage in a wheat-maize intercropping system to increase crop productivity and reduce CEs. The resultant crop yields, water use, solar energy use efficiency (SUE), economic benefits, and CEs of wheat/maize intercropping patterns to various straw returning options were determined in an arid oasis region. We formulated the following hypotheses: (i) intercropping wheat and maize could improve crop productivity when compared with monocultures of either crop under conventional tillage and (ii) reduced tillage and changes to the way straw residue is handled could improve crop productivity of intercrops while reducing CEs.

## Materials and Methods

### Study Site

The experiment was carried out at the Agricultural Research Station (37°34’N, 102°94’E) of Gansu Agricultural University from 2010 to 2012. The research station is located in the eastern part of Hexi Corridor of northwestern China, with the sunshine duration above 2940 h, and accumulated temperature above 10°C greater than 2985°C, in each year. Temperature conditions were suitable for intercropping patterns. The annual mean precipitation of the crop growing season is below 160 mm, but the potential soil water evaporation is greater than 2400 mm. The low water availability limits the potential of cropping extension based on conventional tillage. Thus, plastic mulching and the return of crop straw to the field were applied to crop production to improve water utilization. Water shortage and a gradual shrink in arable land in that area result in a conflict between crop water requirements and food demands. Therefore, an innovative planting pattern for the intercropping system has been introduced to the Hexi Corridor of China. Maize and wheat are the two main grain crops in this region; they are planted in monoculture and intercropping systems. The soil at the experimental site is classified as a desert soil, a kind of desert land filled with calcareous particles. During the two study years, the precipitation during the wheat growing season (March–July) was 65.8 and 40.5 mm and the precipitation during the maize growing season (April–September) was 179.1 mm and 128.5 mm, in 2011 and 2012, respectively.

### Experimental Design

A preparatory experiment was conducted in 2010 to create suitable straw management approaches and to provide a field basis for the implementation of various treatments in 2011 and 2012. In the testing years, three kinds of straw retention patterns were implemented, including plastic mulch applied to maize strips before sowing, forming an advanced pattern with crop straw and plastic mulching (i.e., both straw and plastic were used to mulch the maize strips). The wheat-maize intercropping was tested in three crop-straw retention systems, and conventional tillage treatment without straw residue was applied to monoculture wheat, maize, and wheat-maize intercropping patterns. Thus, there were six treatments with three replicates constituting a total of 18 plots in a randomized, complete block design. Moreover, to improve the intercropping advantage, four straw management approaches were examined: (i) no-tillage with 25–30 cm lengths of straw standing in the plot during the wheat harvesting of the previous summer (NTSSI); (ii) no-tillage with 25–30 cm lengths of straw covering the soil surface during the wheat harvesting of the previous summer (NTSI); (iii) tillage with 25–30 cm lengths of straw incorporated into the soil through deep tillage after wheat harvesting of the previous summer (TSI); and (iv) conventional treatment without straw residue (CTI), such as deep tillage (30 cm depth) with wheat straw removed from the field.

**FIGURE 1:**
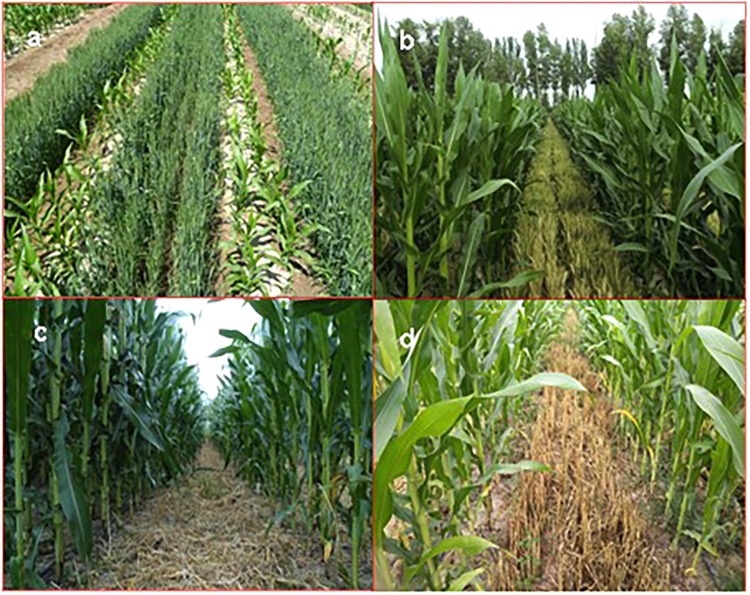
Wheat-maize intercropping systems tested at the Wuwei Research Station, China, with **(A)** the early wheat-maize co-growth stage, **(B)** the late wheat-maize co-growth stage, **(C)** wheat straw of 25–30 cm lengths that were chopped and evenly spread on the soil surface, and **(D)** 25–30 cm lengths of wheat straw standing in the field at the time of wheat harvesting.

The four crop-straw modes were applied to the wheat/maize intercropping patterns (**Figure 1**). The different tillage and stubble retention practices were applied to the wheat strips in various wheat-maize intercropping systems only, and all the maize strips were tilled. Since the main objective is to discover intercrop response to various straw retention options, no single crop was subjected to conservation practices but only for conventional tillage (i.e., only conventional monoculture wheat was rotated with monoculture maize in alternate years) to form conventional monoculture maize treatments (i.e., CTM), and monoculture wheat (i.e., CTW) were included in this study. Conventional tillage without straw retention was used as the control where the soil (30 cm) was plowed the previous fall for weed control; then, an 80 cm wide rotavator was operated at a depth of 30 cm at pre-seeding for seedbed preparation. In late fall of each year, wheat strips were managed to form the different tillage and straw residue options, and maize strips were deeply plowed and raked. In the following spring, the soil was firstly fertilized, harrowed, smoothed, and compacted in the maize-preceded strips; then, a wheat crop was planted in the maize-preceded strips with a strip rotary tillage wheat seeder. At the same time, a plastic film was mulched on the wheat straw surface in the wheat-preceded strips, and maize was planted on the wheat-preceded strips with a manual duckbill punch roller dibbler. For the plastic film mulching on maize strips under the straw standing, a stone roller was applied to compact and crush the standing straw. Then, the surface was mulched with a 90 cm wide plastic film, and its two edges were covered with soil.

For all treatments, maize stalks were removed from the fields for animal feeding. In the experiment conducted the next year, conventional monoculture maize and wheat were alternated among the two years (i.e., the plots grown with monoculture maize the previous year were planted with monoculture wheat the current year and vice versa). In the wheat-maize intercropping, the maize strips from the previous year were planted with wheat in the current year and vice versa, thus, forming an “intra-field strip rotation.” This was implemented to avoid potential weaknesses or problems that might occur with continuous cultivation and to balance soil nutrients that were required by the two different crops in the alternate years.

Field wheat (cv. *Yong-liang 4*) was planted on 20 March and 19 March and was harvested on 22 July and 18 July in 2011 and 2012, respectively. Maize (cv. *Ji-xiang 1*) was planted on 17 April and 20 April and was harvested on 28 September and 2 October, in the two testing years, respectively. The plot size was 48 m^2^ (4.8 m × 10 m). Intercropped wheat was planted in six rows of 80 cm strip width (12 cm row space), and intercropped maize was alternately planted in two rows of 80 cm strip width (40 cm interrow). Planting density was 675 plants m^-2^ (375 kg ha^-1^) for monoculture wheat and 8.25 plants m^-2^ for maize; in the same occupied area, the intercrops’ density was consistent with that of monocultures. Urea and diammonium phosphate were broadcast and then incorporated into the soil for seeding. The N rates applied to maize and wheat were 450 kg ha^-1^ and 225 kg ha^-1^, respectively, and P rates were 225 kg ha^-1^ and 150 kg ha^-1^ in the monoculture maize and wheat, respectively. In the same occupied area, each intercrop received N and P rates consistent with those of monocultures. All N and P were used as base fertilizers for wheat, but for maize crops, 30% of N was applied as base fertilizer, 60% was applied for jointing, and the remaining 10% was applied during the grain-filling stage. Owing to low precipitation at the testing regions, supplemental irrigation was applied using flood irrigation with a hydrant pipe system, and a flow meter was installed at the inlet end to measure and record the amount of irrigation in each plot. All treatments received 120 mm of irrigation water (IW) during late fall, and then, the required irrigation quotas were applied to each cropping pattern during the growing season (**Table 1**).

**Table 1:** Irrigation date and irrigation quotas at the main growth stage of monoculture and wheat-maize intercropping systems in an oasis region in 2011 and 2012.

Irrigation stage	Irrigation date		Irrigation quota		
		
	2011	2012	Monoculture wheat	Monoculture maize	Wheat-maize intercropping
	——-Month-day——–		———————————————–mm——————————————————-		
Wheat jointing stage/maize seedling stage	May-08	May-05	75	-	75
Wheat booting stage/maize jointing stage	May-29	May-26	90	90	90
Wheat filling stage/maize pre-heading stage	Jun-26	Jun-23	75	75	75
Maize silking stage	Jul-25	Jul-21	-	90	90
Maize early-filling stage	Aug-10	Aug-08	-	75	75
Maize mid-filling stage	Aug-27	Aug-22	-	75	75
Total	-	-	240	405	480


### Data Collection

#### Grain Yield (GY) and Land Equivalent Ratio (LER)

All experimental plots were harvested by hand when the crops reached full maturity, and the grains were individually determined based on the air-dried, cleaned crops of different treatments in the monocultures and intercrops.

Land equivalent ratios (LERs) were used in the evaluation of the land use advantage provided by intercropping (Gou et al., 2016), which was calculated using the equation given below:

$$LER={LER}_{wheat}+{LER}_{maize}=\frac{Y_{\mathrm{int}w}}{Y_{monow}}+\frac{Y_{\mathrm{int}m}}{Y_{monom}}$$

where Y_intw_ and Y_intm_ are the yields of intercropped wheat and maize, and Y_monow_ and Y_monom_ are the yields of corresponding monoculture wheat and maize. An LER greater than 1.0 indicates more efficient land use and vice versa.

#### Soil Water Characteristics

##### Water use efficiency (WUE)

Water use efficiency was calculated using grain yield (GY, kg ha^-1^) divided by evapotranspiration (ET, mm) during the entire growing period.

$$WUE=\frac{GY}{ET}$$

The ET was the sum of soil water storage at the sowing stage minus soil water storage at the harvesting stage of crops, plus precipitation and irrigation across the crop growing season. The ET of the various growing periods was the sum of soil water storage at the early stage minus soil water storage at the late stage of crop growth, plus precipitation and irrigation during this growing stage of crops.

The ET modulus coefficient was calculated by ET of various growing periods divided by total ET across the entire growing period.

Soil water content (%) was measured using the oven-drying method. Soil water content in the 0–120 cm soil depth of each crop was measured at an interval of 20 days during the entire growing season. Therefore, it was measured at two points for both wheat and maize strips in the intercropping system, where the soil water content from 0 to 30 cm soil depth was measured in 10 cm increments and from 30 to 120 cm soil depth in 30 cm increments. The soil water content was determined in wheat and maize strips in the intercropping plot and between the two central rows in the monoculture plots. The average value of the two strips was used for the intercropping treatments. Soil water content was also measured prior to and after each irrigation. The volumetric water content (cm^3^ cm^-3^) was obtained by multiplying the gravimetric water content by the soil bulk densities of 1.38, 1.40, 1.45, 1.51, 1.50, and 1.48 g cm^-3^ for the six soil depths. The bulk density was determined using undisturbed soil cores via cutting ring at sowing. The volumetric water content was converted into soil water storage (*SWS*, mm) using the depth of the soil as follows:

SWS=θv×h×10

where θ*_v_* is the volumetric water content at a specific soil layer (cm^3^ cm^-3^), and *h* is the soil depth increment (cm).

##### Water productivity (WP)

Water productivity was calculated as GY (kg ha^-1^) divided by the total IW (mm) and is expressed as follows (Ali et al., 2017):

$$WP=\frac{GY}{IW}$$

#### Solar Energy Use Efficiency (SUE)

In this study, SUE (%) was defined as the ratio of the total energy production based on the grain and biomass yield of crops to the total solar radiation per unit area during the entire crop growing period, and the equation was expressed as follows:

$$SUE=\frac{hm}{Q}$$

where ‘’h’ is the heat production rate (16.3 MJ kg^-1^ for the grain of wheat and maize and 14.6 MJ kg^-1^ for the biomass of wheat and maize) (Carroll et al., 1990; Chai et al., 2014), ‘m’ is the yield of grain or biomass, and ‘Q’ is the total solar radiation per unit area, which was determined using an automatic weather station (SL5) during the entire crop growing period.

#### Economic Analysis

##### Total output value (TOV)

The total output value (TOV) was calculated using the following equation:

$$TOV=P_{ws}*Y_{ws}+P_{wg}*Y_{wg}+P_{ms}*Y_{ms}+P_{mg}*Y_{mg}$$

where P and Y denote the prices and yields for crops, the subscripts are ‘ws’ for wheat straw, ‘wg’ for wheat grain, ‘ms’ for maize straw, and ‘mg’ for maize grain, for each cropping pattern, respectively. In the two testing years, the prices of grain and straw for wheat was 2.4 and 0.6 ¥ kg^-1^; the prices of grain and straw for maize was 1.8 and 0.2 ¥ kg^-1^, respectively. The price expressed in USD was based on the exchange rate of 634.8 ¥ for every 100 USD in 2011 and 632.5 ¥ for every 100 USD in 2012.

##### Net return (NR)

The net return (NR) is defined as the difference in the TOV and the total cost, and the equation is as follows:

NR=TOV−TC

where TOV is the total output value and TC is the total variable cost, which includes labor and machine costs as well as the costs of planting materials, fertilizer, plastic film, and irrigation in this experiment. However, the labor cost is treated as a constant in this study, because an almost equal amount of time was spent on each treatment. The number of hours spent clearing, weeding, and applying fertilizer were assumed to be equal in all treatments. During the two study years, the price of irrigation was 0.3 ¥ m^-3^; the prices of urea, diammonium phosphate, and plastic film were 1.65 ¥, 3.5 ¥, and 13 ¥ kg^-1^; the prices of wheat and maize seeds were 6 ¥ and 26 ¥ kg^-1^, respectively. The cost of seedbed preparation (such as fertilized, harrowed, smoothed, and compacted) was 450 ¥ ha^-1^. The cost of cultivated land after wheat harvest was 750 ¥ ha^-1^. Wheat was harvested, threshed, and transported by hand, and the cost was 4500 ¥ ha^-1^; maize was harvested, threshed and transported by machine, and the cost was 1500 ¥ ha^-1^.

##### Benefit per cubic meter of water (BPW)

The benefit per cubic meter (BPW) of water was determined by dividing the NR by the total ET and is expressed as follows:

$$BPW=\frac{NR}{ET}$$

#### Carbon Emission Characteristics

##### Soil respiration

Soil respiration (R_S_) was measured using a CFX-2 system (Soil CO_2_ Flux System, CFX-2, PP System Hitchin, United Kingdom) connected to a proprietary respiration chamber. The chamber was pushed gently into the hole to a depth of 3 cm in the center of each crop of monocultures and intercropped wheat and maize strips. For monocultures, the measuring value of each crop was used for a plot. For intercropping, the measuring values were taken for each crop strip, and the average value of the two strips were used for a plot (Hu et al., 2015). During the entire crop growing season, soil respiration was monitored at 15-day intervals and every 2 h from 8:00 am to 8:00 pm on the measuring dates; soil respiration from 9:00 am to 12:00 am could minimize diurnal variation in flux patterns and could represent averaged soil respiration during the whole day (24 h) (Zhai et al., 2011).

##### Carbon emissions (CE)

Carbon emission (kg ha^-1^) was calculated using the following equation (Zhai et al., 2011):

$$CE=\Sigma \left[\frac{Rs(i+1)+Rsi}{2}[t(i+1)-ti]\times \frac{12}{44}\right]\times 24\times 10$$

where Rs is soil respiration (g CO_2_ m^-2^ hr^-1^), ‘i + 1’ and ‘i’ are the current and the last measuring date, respectively, and ‘t’ is days after the sowing stage. The numbers 10 and 24 are the conversion factors of the numerical unit of CE from g CO_2_ m^-2^ hr^-1^ to kg ha^-1^.

##### Carbon emission efficiency (CEE)

To quantify the relationship between GY and CEs, carbon emission efficiency (CEE) was calculated using the following equation:

$$CEE=\frac{GY}{CE}$$

where GY and CE are grain yield (kg ha-1) and CEs (kg ha^-1^), respectively.

### Statistical Analysis

The data were analyzed using the Statistical Package for the Social Sciences statistical analysis software (SPSS software, 19.0; SPSS Inst. Ltd., United States). Two-way analysis of variance (ANOVA) followed by Duncan’s multiple-range test was performed to determine the treatment effects (straw retention approaches) and year × treatment interactions for most of the variables assessed.

## Results

### Crop Yields and Land Equivalent Ratio

**FIGURE 2:**
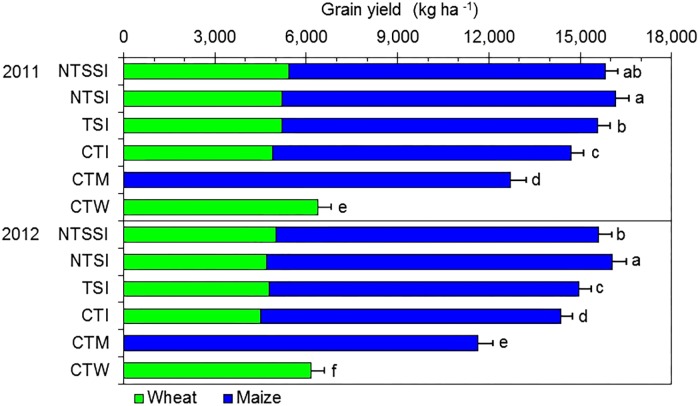
Grain yield of wheat and maize in monoculture and wheat-maize intercropping systems under different straw retention approaches in 2011 and 2012. Different letters indicate significant differences (*p* < 0.05) among treatments within a year. The error bars indicate the standard errors of the means (*n* = 3). The descriptions of the treatment names: NTSSI, intercropping using no-tillage with straw standing; NTSI, intercropping using no-tillage with straw covering; TSI, intercropping using tillage with straw incorporated into the soil; CTI, intercropping using conventional tillage without straw retention; CTW, monoculture wheat using conventional tillage; and CTM, monoculture maize using conventional tillage without straw retention.

There was a significant effect of year × treatment interaction on crop yield, and straw retention approaches individually had a significant effect on GY of intercropping systems in each year (**Figure 2**). Wheat/maize intercropping patterns increased GY by 16–38% and 130–160%, respectively, compared with conventional monoculture maize and wheat. In particular, NTSI treatment boosted GY by 27 and 38% in 2011 and 2012, compared with CTM treatment; more noticeably, NTSI treatment boosted GY by 153 and 160% compared with CTW treatment, respectively.

Total GYs represent the sum of the intercropped maize and wheat in the strip intercropping patterns (**Figure 2**). Wheat straw residue retention applied to intercropped treatments produced significantly higher GYs than the CTI treatment (12.0 and 5.6%, respectively, in 2011 and 2012). In particular NTSI boosted GY by 9.9% in 2011 and by 11.9% in 2012 compared with CTI treatment.

Straw retention significantly increased the maize GY but had little or no effect on wheat GY in wheat-maize intercropping patterns (**Figure 2**). On average, the GYs of maize under NTSSI, NTSI, and TSI treatments were 7.0, 13.8, and 4.6% higher than those of the CTI treatment. Meanwhile, intercropped maize yields accounted for 65.7–70.8% of the total GYs, indicating that maize crop is the major contributor in wheat-maize intercropping patterns.

**FIGURE 3:**
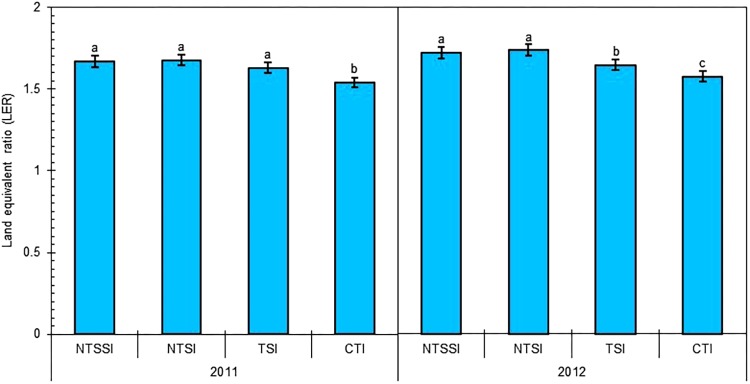
Land equivalent ratio (LER) of wheat-maize intercropping systems under different straw retention approaches in 2011 and 2012. Different letters indicate significant differences (*p* < 0.05) among treatments within a year. The error bars indicate the standard errors of the means (*n* = 3). The descriptions of the treatment names: NTSSI, intercropping using no-tillage with straw standing; NTSI, intercropping using no-tillage with straw covering; TSI, intercropping using tillage with straw incorporated into the soil; CTI, intercropping using conventional tillage without straw retention; CTW, monoculture wheat using conventional tillage; and CTM, monoculture maize using conventional tillage without straw retention.

Using the LER to indirectly assess the level of intercropping advantage to increase yield over monoculture, we found that the LERs of the wheat/maize intercropping (averaging to 1.63 in 2011 and 1.67 in 2012) was greater than 1.0 (**Figure 3**). The result indicated that this cropping system used less land but produced more grain than the corresponding monocultures. The NTSSI, NTSI, and TSI treatments significantly improved the LER of wheat/maize intercropping from 8.5% to 9.2%, 9.0% to 10.1%, and 4.5% to 5.9% in comparison with conventional tillage treatment, respectively.

### Evapotranspiration and Evapotranspiration Modulus Coefficient

The year × treatment interaction was significant for ET and ET modulus coefficient of various treatments most likely due to the complex weather conditions (**Table 2**). Wheat-maize intercropping patterns had greater total ET than monoculture maize and wheat during the entire growing period. Among the four intercropping treatments, NTSI treatment reduced total ET by 3.7–4.5% compared to CTI treatment. This suggests that the wheat straw covering measure applied to intercropping pattern is one of the most fundamental farming practices for saving water during agricultural production.

**Table 2:** Evapotranspiration (ET) and evapotranspiration modulus coefficient (EC) of crops at each growth stage in monoculture and wheat-maize intercropping systems under different straw retention approaches in an oasis region in 2011 and 2012.

Year	Treatment^a^	P1—P2^b^		P2—P3		P3—P4		P4—P5		P5—P6		P6—P7		Total ET (mm)
								
		ET^c^ (mm)	EC (%)	ET (mm)	EC (%)	ET (mm)	EC (%)	ET (mm)	EC (%)	ET (mm)	EC (%)	ET (mm)	EC (%)	
**2011**	**Intercropping**												
	NTSSI	32d^d^	4.2c	99c	13.1c	120ab	15.9c	128bc	17.0cd	95c	12.6c	280ab	37.3b	752ab
	NTSI	28e	3.9d	96c	13.0c	117b	15.8c	130b	17.7bc	78d	10.5d	288a	39.1a	736b
	TSI	36c	4.7b	106b	13.95b	113bc	14.9d	123bc	16.2d	113a	14.9b	268c	35.4c	756ab
	CTI	36b	4.7b	107b	13.93b	114bc	14.8d	125bc	16.2d	116a	15.1b	273bc	35.3c	771a
	Monoculture												
	CTM	—	—	86d	13.3b	110c	17.1b	116d	18.0b	107b	16.5a	227d	35.2c	644c
	CTW	40a	9.4a	117a	27.53a	125a	29.5a	143a	33.7a	—	—	—	—	424d
**2012**	**Intercropping**												
	NTSSI	40c	5.9c	84cd	12.5d	100b	14.8bc	117b	17.4c	72c	10.6c	262a	38.8a	676bc
	NTSI	36d	5.4d	81d	12.0d	101b	15.0b	117b	17.5c	66d	9.8d	269a	40.2a	670c
	TSI	45a	6.5b	94b	13.7c	98bc	14.3c	113bcd	16.5d	83b	12.1b	253ab	36.8b	686ab
	CT	46a	6.6b	98b	14.1bc	102ab	14.7bc	116bc	16.7cd	85ab	12.3b	248b	35.7b	695a
	**Monoculture**												
	CTM	—	—	85c	14.4b	92c	15.6b	108d	18.3b	92a	15.5a	214c	36.2b	592d
	CTW	40b	10.6a	102a	27.1a	108a	28.4a	128a	33.9a	—	—	—	—	378e


*^*a*^NTSSI, intercropping using no-tillage with straw standing; NTSI, intercropping using no-tillage with straw covering; TSI, intercropping using tillage with straw incorporated into the soil; CTI, intercropping using conventional tillage without straw retention; CTW, monoculture wheat using conventional tillage; CTM, monoculture maize using conventional tillage without straw retention. ^*b*^P1–P7 indicates the various crop growing periods. The sampling dates were March 27, April 16, May 20, June 20, July 22, August 10, September 28 in 2011 and March 18, April 19, May 21, June 20, July 18, August 11, October 2 in 2012. The corresponding growing periods of maize were pre-sowing (wheat sowing), sowing (wheat seeding), jointing (wheat boosting), pre-heading (wheat filling), silking (wheat harvesting), early-filling, and harvesting, respectively. ^*c*^ET, evapotranspiration; EC, evapotranspiration modulus coefficient, which was calculated by evapotranspirations of various growing periods divided by total evapotranspiration across the entire growing period. ^*d*^Values followed by different letters in the same column of the same year are significantly different at *p* < 0.05.*

From the wheat sowing stage to the maize jointing stage (responding to wheat booting stage), NTSSI and NTSI treatments reduced ET by 7.5–20.0% and 9.2–28.3% compared with CTW treatment, respectively (**Table 2**). Similarly, the ET modulus coefficient was reduced by 44.8–54.9% and 48.8–58.7%, respectively. Among the four intercropping treatments, NTSSI and NTSI had lower ET and ET modulus coefficient, compared with CTI treatment, and reduced ET by 8.3–13.7% and 11.1–21.6%, respectively. Similarly, these two treatments reduced the ET modulus coefficient by 6.0–11.1% and 6.9–17.8%, respectively.

From the maize jointing stage to the silking stage (responding to the wheat booting stage to the harvesting stage), NTSSI and NTSI treatments reduced ET by 4.3–10.6% and 6.3–8.7% compared with CTW treatment, respectively (**Table 2**). Similarly, these two treatments decreased the ET modulus coefficient by 46.1–49.6% and 46.3–48.5%, respectively. Compared with CTM treatment, ET was increased by 8.3–12.1% under NTSSI treatment, by 6.1–12.6% under NTSI treatment, by 4.3–10.0% under TSI treatment, and by 6.7% to 14.4% under CTI treatment. However, the ET modulus coefficient was reduced by 6.6–12.8% and 4.2–13.6% under TSI and CTI treatments, respectively. After wheat harvest and from the maize silking stage to the early filling stage, NTSSI and NTSI treatments lowered ET by 11.2–18.4% and 24.9–27.1% compared with CTM treatment, respectively. Similarly, these two treatments decreased the ET modulus coefficient by 23.9–29.6% and 34.7–36.3%, respectively. Among the four intercropping treatments, the ET of NTSSI and NTSI treatments were reduced by 16.2–18.6% and 22.9–33.3%; similarly, ET modulus coefficient were reduced by 13.7–16.6% and 20.0–30.1% compared with CTI treatment, respectively. Additionally, compared with TSI treatment, NTSSI and NTSI treatments reduced ET by 14.0–16.0% and 20.8–31.1% and reduced ET modulus coefficient by 12.6–15.5% and 18.9–29.2%, respectively.

However, from the maize early filling stage to the harvesting stage, the intercropping pattern had a greater ET and ET modulus coefficient than conventional monoculture maize (**Table 2**). Compared with CTM treatment, NTSSI and NTSI treatments increased ET by 23.6–24.0% and 27.0–27.5% and increased the ET modulus coefficient by 6.0–6.9% and 10.7–12.3%, respectively. Across the four intercropping treatments, NTSSI and NTSI treatments increased ET by 3.0–5.5% and 5.7–8.5%, compared with CTI treatment; similarly, these two treatments also increased the ET modulus coefficient by 5.4–8.6% and 10.7–12.5%, respectively. Additionally, NTSSI and NTSI treatments increased ET by 3.9–4.7% and 6.7–7.7% and increased the ET modulus coefficient by 5.3–5.6% and 9.3–10.6% compared with TSI treatment, respectively.

In other words, the NTSSI and NTSI treatments reduced ET of the intercrop before the maize silking stage but increased it afterward. This created a more optimal balance between early- and late-stage intercrop water demand. The effect of NTSI treatment was the best.

### Water Use Efficiency and Water Productivity

**FIGURE 4:**
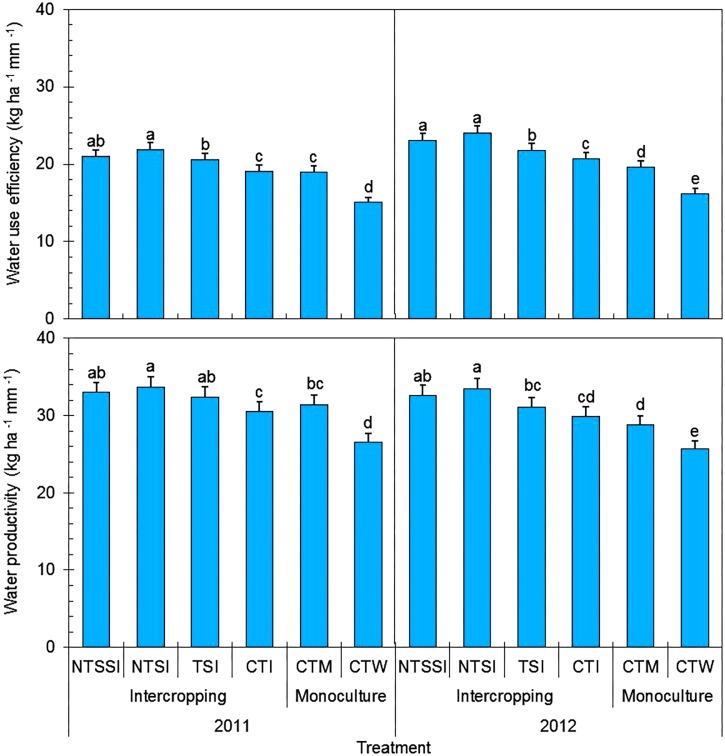
Water use efficiency and water productivity of monoculture and wheat-maize intercropping systems under different straw retention approaches in 2011 and 2012. Different letters indicate significant differences (*p* < 0.05) among treatments within a year. The error bars indicate the standard errors of the means (*n* = 3). The descriptions of the treatment names: NTSSI, intercropping using no-tillage with straw standing; NTSI, intercropping using no-tillage with straw covering; TSI, intercropping using tillage with straw incorporated into the soil; CTI, intercropping using conventional tillage without straw retention; CTW, monoculture wheat using conventional tillage; and CTM, monoculture maize using conventional tillage without straw retention.

The year × treatment interaction was significant for WUE. However, the trend of the treatment effects was similar across the three study years (**Figure 4**). The wheat/maize intercropping with wheat straw retention significantly improved WUE in comparison with the conventional monoculture systems. The straw residue retention that was applied to intercropping patterns improved WUE by 8.6–22.4% and 34.6–48.3% compared with conventional monoculture maize and wheat treatments, respectively. In particular, NTSI treatment had greater WUE than the CTM treatment by 15.4% in 2011 and 22.4% in 2012. More dramatically, NTSI treatment had greater WUE than the CTW treatment by 45.7% in 2011 and 48.3% in 2012. Across the four intercropping treatments, NTSI had greater WUE than CTI by 14.7% in 2011 and 15.9% in 2012.

Similarly, there was a significant effect of year × treatment interaction on WP, but the trend of the treatment effects was similar across the three study years (**Figure 4**). Wheat-maize intercropping, coupled with wheat straw residue retention, significantly improved WP by 3.4–16.4% and by 21.1–30.2% in comparison with the conventional monoculture maize and wheat treatments, respectively. In particular, the NTSI treatment increased WP by 7.3% in 2011 and 16.4% in 2012 compared with the CTM treatment, by 26.6% in 2011 and 30.2% in 2012 compared with the CTW treatment, and by 9.9% in 2011 and 12.0% in 2012 compared with the CTI treatment.

### Solar Energy Use Efficiency

**FIGURE 5:**
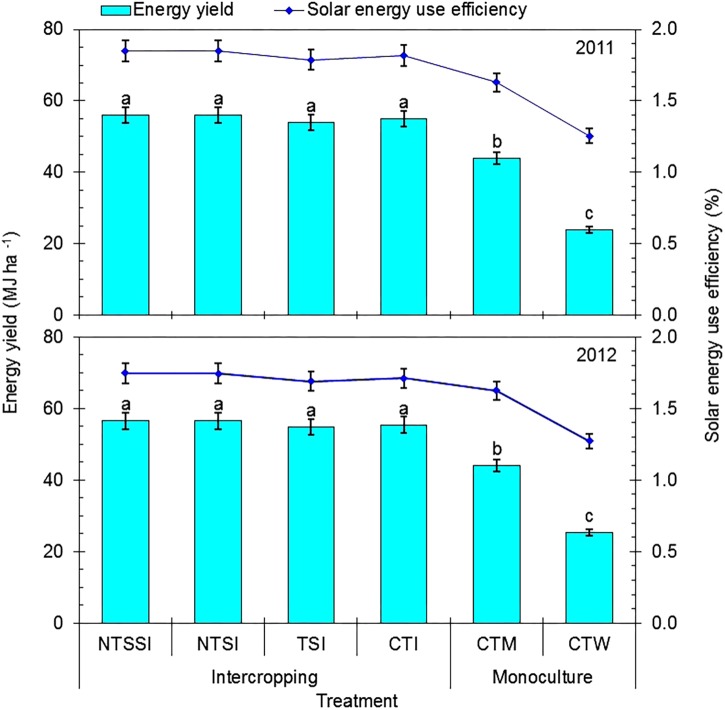
Energy yields and solar energy use efficiencies of monoculture and wheat-maize intercropping systems under different straw retention approaches in 2011 and 2012. Different letters indicate significant differences (*p* < 0.05) among treatments within a year. The error bars indicate the standard errors of the means (*n* = 3). The descriptions of the treatment names: NTSSI, intercropping using no-tillage with straw standing; NTSI, intercropping using no-tillage with straw covering; TSI, intercropping using tillage with straw incorporated into the soil; CTI, intercropping using conventional tillage without straw retention; CTW, monoculture wheat using conventional tillage; and CTM, monoculture maize using conventional tillage without straw retention.

There was no significant effect of year × treatment interaction on energy yields, but straw retention approaches had a significant effect on energy yields in each year (**Figure 5**). Wheat-maize strip intercropping had energy yields of 120.4–131.0% and 25.8–26.7% more than those of conventional monoculture wheat and maize, respectively. The NTSSI, NTSI, TSI and CTI treatments boosted energy yields by 123.3–134.1%, 123.1–134.0%, 116.2–125.9%, and 118.9–129.6% in comparison with CTW treatment and by 27.6–28.3%, 27.6–28.2%, 23.1–24.3%, and 25.1–25.9% in comparison with CTM treatment, in the two study years, respectively. However, there was no significant difference in energy yields among the four intercropping treatments.

Similarly, there was no significant effect of year × treatment interaction on SUE, but straw retention approaches had a significant effect on SUE in each year (**Figure 5**). Wheat-maize strip intercropping had SUEs of 35.3–45.6% and 6.1–12.2% (with a mean value of 1.78%) more than those of conventional monoculture of wheat and maize, respectively. Importantly, NTSSI and NTSI treatments increased SUE by 37.1–47.6% and 37.0–37.7% in comparison with CTW treatment and by 7.5–13.7% and 7.4–13.7% in comparison with CTM treatment, in the two study years, respectively. However, there was no significant difference in SUEs among the four intercropping treatments.

### Economic Benefits Analysis

#### Output Value and Net Return

There was a significant effect of year × treatment interaction on output value (**Table 3**). The wheat-maize intercropping system obtained a comparatively higher TOV, averaging to 6,121 USD ha^-1^ annually, which was 26% higher (1,277 USD ha^-1^) than CTM and 131% higher (3,473 USD ha^-1^) than CTW. The NTSI treatment was the most productive pattern and increased the TOV by 28% (1,330 USD ha^-1^) in 2011 and 34% (1,687 USD ha^-1^) in 2012 compared with the CTM treatment. Similarly, and more noticeably, NTSI treatment increased the TOV by 133% (3,470 USD ha^-1^) in 2011 and by 146% (3,941 USD ha^-1^) in 2012 compared with the CTW treatment. Moreover, the NTSI treatment had higher TOVs, which were 6% higher (325 USD ha^-1^) in 2011 and 8% higher (495 USD ha^-1^) in 2012 than the CTI treatment.

**Table 3:** Total output value and net return of wheat and maize in monoculture and wheat-maize intercropping systems under different straw retention approaches in an oasis region in 2011 and 2012.

Year	Treatment^a^	Cost								Total output value	Net return
				
		Labor	Machine	Herbicide	Fertilizer and straw	Plastic film	Irrigation	Seeds	Total		
	—————————————————————–USDha^-1^—————————————————————–										
**2011**	**Intercropping**										
	NTSSI	634	109	34	485	65	284	276	1,887ab^b^	5,975a	4,088a
	NTSI	617	109	28	479	65	284	276	1,858b	6,074a	4,216a
	TSI	656	132	40	477	65	284	276	1,931b	5,708b	3,777b
	CTI	682	132	53	367	65	284	276	1,860b	5,749b	3,889b
	**Monoculture**										
	CTW	455	132	55	288	—	170	397	1,497c	2,604d	1,107d
	CTM	845	132	47	447	130	248	156	2,005a	4,744c	2,739c
**2012**	**Intercropping**										
	NTSSI	555	109	35	504	71	285	304	1,862bc	6,480a	4,618b
	NTSI	534	109	29	502	71	285	304	1,833c	6,632a	4,800a
	TSI	576	133	41	498	71	285	304	1,907b	6,213b	4,306c
	CTI	605	133	55	383	71	285	304	1,834c	6,137b	4,303c
	**Monoculture**										
	CTW	498	133	57	300	—	171	436	1,595d	2,691d	1,097e
	CTM	925	133	49	465	142	249	171	2,134a	4,945c	2,811d


*^*a*^NTSSI, intercropping using no-tillage with straw standing; NTSI, intercropping using no-tillage with straw covering; TSI, intercropping using tillage with straw incorporated into the soil; CTI, intercropping using conventional tillage without straw retention; CTW, monoculture wheat using conventional tillage; CTM, monoculture maize using conventional tillage without straw retention. ^*b*^Values followed by different letters in the same column of the same year are significantly different at *p* < 0.05.*

Similarly, a significant year × treatment interaction affected NR, and straw retention significantly improved NR for intercropping treatments (**Table 3**). The wheat-maize intercropping pattern obtained a comparatively higher NR, averaging to 4,249 USD ha**^-^**^1^ annually, which was 53% (4249 vs. 2774 USD ha**^-^**^1^) and 286% (4249 vs. 1102 USD ha**^-^**^1^) greater than those of CTM and CTW treatments, respectively. The NTSI treatment increased the NR by 54% (4216 vs. 2739 USD ha**^-^**^1^) in 2011 and by 71% (4800 vs. 2811 USD ha**^-^**^1^) in 2012 compared with the CTM treatment. Similarly, NTSI treatment increased the NR by 281% (4216 vs. 1107 USD ha**^-^**^1^) in 2011 and by 338% (4800 vs. 1097 USD ha**^-^**^1^) in 2012 compared with the CTW treatment. Moreover, the NTSI treatment increased the NR by 8% (4216 vs. 3889 USD ha**^-^**^1^) in 2011 and by 14% (4800 vs. 4303 USD ha**^-^**^1^) in 2012 in comparison with the CTI treatment.

The fact that wheat-maize intercropping produced a higher NR relative to conventional monocultures could be attributed to its higher TOV and its lower total production costs, averaging to an annual 198 USD ha**^-^**^1^ lower than conventional monocultures. Across wheat-maize intercropping systems, straw standing and straw covering produced comparatively higher NRs in comparison with intercropping without straw residue retention, which was attributed to the higher TOV of the straw standing and straw covering treatments.

#### Benefit per Cubic Meter of Water (BPW)

**FIGURE 6:**
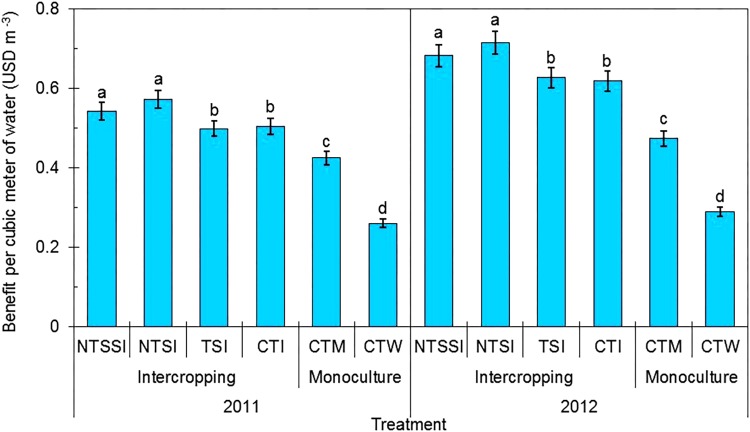
Benefit per cubic meter of water in monoculture and wheat-maize intercropping systems under different straw retention approaches in 2011 and 2012. Different letters indicate significant differences (*p* < 0.05) among treatments within a year. The error bars indicate the standard errors of the means (*n* = 3). The descriptions of the treatment names: NTSSI, intercropping using no-tillage with straw standing; NTSI, intercropping using no-tillage with straw covering; TSI, intercropping using tillage with straw incorporated into the soil; CTI, intercropping using conventional tillage without straw retention; CTW, monoculture wheat using conventional tillage; and CTM, monoculture maize using conventional tillage without straw retention.

There was a significant effect of year × treatment interaction on BPW (**Figure 6**). However, the trend of the treatment effects was similar across the three study years. A higher BPW was observed in wheat-maize intercropping systems, which were 32% greater than that of CTM and 116% greater than that of CTW. The NTSI treatment was the best treatment in this regard and increased BPW by 35% in 2011 and by 51% in 2012 in comparison with CTM treatment. Similarly, BPW under the NTSI treatment was increased by 119 and 147% in 2011 and 2012, respectively, in comparison with the CTW treatment and by 14 and 16%, respectively, in comparison with the CTI treatment. Wheat straw covering in intercrops is one of the most effective strategies to increase crop productivity, improve NR, and increase BPW in developing a sustainable agricultural system.

### Carbon Emission Characteristics

#### Carbon Emissions During the Growing Season

**FIGURE 7:**
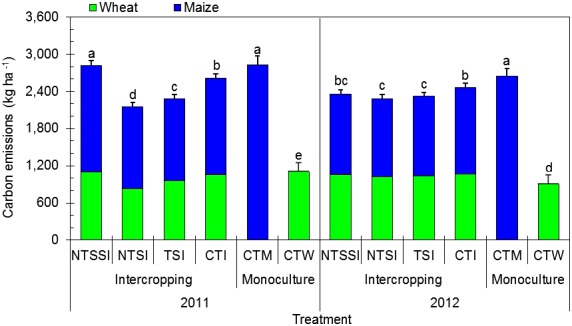
Carbon emissions of monoculture and wheat-maize intercropping systems under different straw retention approaches in 2011 and 2012. Different letters indicate significant differences (*p* < 0.05) among treatments within a year. The error bars indicate the standard errors of the means (*n* = 3). The descriptions of the treatment names: NTSSI, intercropping using no-tillage with straw standing; NTSI, intercropping using no-tillage with straw covering; TSI, intercropping using tillage with straw incorporated into the soil; CTI, intercropping using conventional tillage without straw retention; CTW, monoculture wheat using conventional tillage; and CTM, monoculture maize using conventional tillage without straw retention.

There was a significant effect of year × treatment interaction on CE during the growing season (**Figure 7**). Wheat and maize intercropping patterns emitted comparatively lower CO_2_ than conventional monoculture maize by 12.8 and 10.8% in 2011 and 2012. In addition, the NTSI treatment significantly reduced CEs by 23.8% in 2011 and 13.4% in 2012, compared with CTM treatment.

Across the intercropping treatments, NTSI and TSI treatments reduced CO_2_ emissions by 7.3–17.5% and 6.0–12.5%, respectively, in comparison with CTI treatment (**Figure 7**). However, NTSSI treatment had no consistent effect on CEs. In terms of CEs in each strip of the intercropping systems, maize strips emitted 22.9–59.7% more carbon than the wheat strips, showing that maize strips were the major contributors of CEs in the entire intercropping pattern.

#### Carbon Emission Efficiency

**FIGURE 8:**
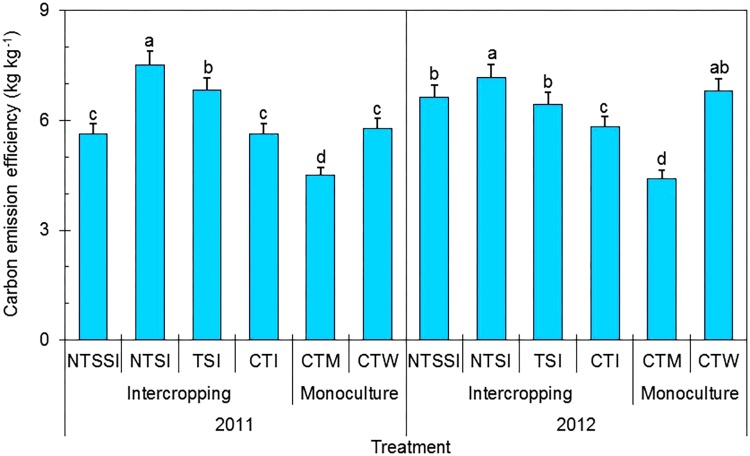
Carbon emission efficiency of monoculture and wheat-maize intercropping systems under different straw retention approaches in 2011 and 2012. Different letters indicate significant differences (*p* < 0.05) among treatments within a year. The error bars indicate the standard errors of the means (*n* = 3). The descriptions of the treatment names: NTSSI, intercropping using no-tillage with straw standing; NTSI, intercropping using no-tillage with straw covering; TSI, intercropping using tillage with straw incorporated into the soil; CTI, intercropping using conventional tillage without straw retention; CTW, monoculture wheat using conventional tillage; and CTM, monoculture maize using conventional tillage without straw retention.

There was a significant year × treatment interaction affected the CEE during the growing season, and straw retention individually had a significant effect on the CEE in each year (**Figure 8**). Wheat-maize intercropping patterns produced a mean CEE value of 6.5 kg kg**^-^**^1^, which was 39.9% greater than that of monoculture maize without straw residue (i.e., CTM). Among the four strip intercropping treatments, NTSI had the highest CEE and improved CEE by 66.9% in 2011 and by 62.6% in 2012 compared with CTM treatment. Similarly, CEE was improved by 30.2% in 2011 and by 5.4% in 2012 compared with CTW treatment and was improved by 33.2% in 2011 and by 23.2% in 2012 compared with CTI treatment. In general, reduced tillage with straw covering was the optimal option for crop straw management approaches to promote CEE of the wheat-maize strip intercropping.

## Discussion

Food security and the reduction of greenhouse gas emissions are two major topics in agricultural research. Some researchers are interested in increasing awareness regarding grain security and the reduction of CEs. However, producers are most interested in NRs and investigations regarding how conventional farming systems can be improved to obtain sufficient food while simultaneously alleviating potentially negative impacts on the environment and increasing economic benefits (Chen et al., 2011). In heavily populated areas that experience natural resource shortages, especially the Hexi Corridor of northwest China, this question needs to be urgently solved. Many studies demonstrate that each agricultural practice has its own effects on crop production, water use, and CEs. This study found that reduced tillage, combined with wheat straw residue strategies applied to wheat-maize intercropping patterns, significantly boosted crop yields, improved water utilization in an arid environment, increased NRs, and reduced CEs from intensified cropping systems consistently across the two testing years.

Intercropping has been introduced in many regions around the world to boost or stabilize crop productivity (Cortésmora et al., 2010; Qin A. et al., 2013) and to balance current crop output with human demands (Mueller et al., 2012). Intercropping also produces higher GY by increasing the effective duration of crop growth because wheat is sown 30 days before maize, which keeps growing past the time of wheat harvest (Li et al., 2001). Meanwhile, advantages of intercropping yield over monocultures have been documented to be caused by improved light conditions, reduced pressures from diseases and weeds (Fernández-Aparicio et al., 2010; Qin J. et al., 2013), enhanced facilitative effects of the intercrops across the co-growth period (Li et al., 2001), and improved GYs and NRs (Abu-Bakar et al., 2014). Among water conservation approaches evaluated in this study, reduced tillage combined with wheat straw covering (i.e., the NTSI pattern) increased WUE and WP, which was mainly attributed to lower soil evaporation and optimized water balance (Yin et al., 2015). Additionally, NTSI treatment reduced ET of the intercrop before the maize silking stage but increased it afterward. This created a more optimal balance between early- and late-stage intercrop water demand. The results from the two year consistently found that wheat straw that was returned to the intercropping pattern significantly increased crop yield, WUE, and WP in comparison with the conventional monocultures.

An integrated, improved cropping pattern can boost crop productivity while reducing the environmental impacts of farmland by substantially decreasing greenhouse gas emissions (Brock et al., 2012; Gan et al., 2014). Beneficial management practices can reduce CEs from farmland by applying proper cropping systems and cultivation measures, such as strip intercropping and crop straw retention based on no-tillage and reduced tillage (Hu et al., 2015). A cropping system is the main component of agricultural production management on farmland through time and space. Meanwhile, using crop rotation is an important strategy in alleviating greenhouse gas emissions from agricultural production (Gan et al., 2011b). Reduced tillage, with crop straw retention, applied to intercropping patterns was found to reduce CEs by effectively decreasing soil respiration (Hu et al., 2015). This is because (a) reduced tillage can decrease soil disturbance, thus, reducing emissions of CO_2_ compared with conventional deep tillage (Fuentes et al., 2012), and (b) crop straw covering retention creates a barrier on the soil surface, helping to reduce CEs (Fuentes et al., 2012). Therefore, an important idea in decreasing CEs from farmland is to use advanced agricultural practices of crop production (Gan et al., 2011a). In terms of this paper, a cropping system based on wheat/maize strip intercropping and reduced tillage, combined with straw retention, can be used to enhance crop productivity and reduce CEs, which is a novel approach. The present study clearly shows that wheat/maize strip intercropping pattern, along with reduced tillage combined with straw covering to create an integrated system, is an effective cropping pattern. Our results clearly show that reduced tillage based on straw covering retention serves as an ideal practice to be integrated with wheat-maize strip intercropping to increase system productivity while alleviating the effects of CEs on the environment and significantly enhancing CEE.

Intercropping has many advantages in terms of better use of environmental resources (Black and Ong, 2000). Efficient resource utilization in intercropping systems is highly dependent on agricultural management techniques, mainly the temporal and spatial distributions of intercrops in the intercropping system (Batugal et al., 1990). Although the high productivity of intercropping systems is often explained by an improvement in light interception and SUEs (Zhang et al., 2008), there is no available academic information regarding solar energy utilization in wheat-maize strip intercropping patterns with straw residue and reduced tillage strategies. The integration of reduced tillage, combined with straw retention, into wheat-maize intercropping patterns was shown to increase SUE by 7.4–13.7% and 37.0–47.7% compared with conventional monoculture of maize and wheat, respectively. This result shows that it is feasible to use this integrated, intercropping system to enhance the use of environmental resources.

Producers are most interested in economic benefits, and an increase in the TOV is the basis for obtaining the highest economic benefit. Strip intercropping has played a very crucial role in solving the problem of insecure grain supplies and in increasing the income of farmers in China (Chai et al., 2014; Sun et al., 2014). Moreover, reduced tillage or no-tillage can lower operation times in the field, thereby, reducing production costs for labor, fuel, machinery, and other equipment while increasing the yield of agricultural production (Raper et al., 1994; Boeckx et al., 2011; Fan et al., 2013). The present study suggests that the integration of reduced tillage, combined with straw covering, into wheat-maize intercropping can improve the TOV (by 28–146%), the NR (by 54–338%), and the BPW (by 35–147%), compared with conventional monoculture treatments. Although the market prices of crop inputs and harvested grain tend to change over the years, the output value of the intercropping pattern with straw covering based on reduced tillage was greater than that of the conventional pattern, which has the same prices of crops. Our research results provide strong evidence that the adaptation of an intensified, improved cropping pattern will alleviate water shortage and CE issues in oasis agricultural regions while increasing the income of farmers.

## Conclusion

Cropping intensification through wheat and maize strip intercropping can significantly boost GYs, improve water utilization, and increase economic return while effectively reducing CEs. This leads to higher WUE and CEE in comparison with conventional monoculture of wheat and maize. Among the approaches evaluated in this study, straw covering the soil surface integrated into strip intercropping (i.e., NTSI) was the most effective pattern in boosting crop productivity. The NTSI treatment increased NR by 54–71% and 281–338% and increased the BPW by 35–51% and 119–147%, in comparison with the conventional monoculture of maize and wheat, respectively. Similarly, the NTSI pattern increased NR and BPW by 8–14% and 14–16% in comparison with the conventional intercropping treatment. Meanwhile, NTSI treatment reduced CEs by 13.4–23.8% and 7.3–17.5%, while improving the CEE by 62.6–66.9% and 23.2–33.2%, compared with the conventional monoculture of maize. We conclude that the reduced tillage-based straw coverage strategy integrated into the intercropping pattern can be used to effectively enhance environmental and economic benefits in arid irrigation areas (arid irrigation areas, that is to say, arid areas that are dependent on irrigation for crop production because of low precipitation).

## Author Contributions

QC and WY conceived and designed the experiment. WY, QC, and FF performed the statistical analyses. WY, YG, FH, ZF, CZ, and AY were involved in field data collection. All authors contributed to writing the paper.

## Conflict of Interest Statement

The authors declare that the research was conducted in the absence of any commercial or financial relationships that could be construed as a potential conflict of interest.
